# Cardiac metastasis causes paradoxical malignant embolism

**DOI:** 10.1002/cnr2.1513

**Published:** 2021-07-15

**Authors:** Britta Janina Wagner, Hans‐Peter Hobbach, Anastasia Janina Hobbach, Lena Katharina Hieggelke, Martin Grond, Nadejda Monsefi, Reinhard Buettner

**Affiliations:** ^1^ Institute of Pathology, University of Cologne, Faculty of Medicine University Hospital Cologne Cologne Germany; ^2^ Department of Cardiology, Angiology and Internal Intensive Care Hospital of Siegen Siegen Germany; ^3^ Department of Cardiology I University Hospital Muenster Muenster Germany; ^4^ Department of Neurology and Neurological Geriatric Medicine Hospital of Siegen Siegen Germany; ^5^ Department of Cardiothoracic Surgery, Helios Heart Center NRW, Siegburg‐Wuppertal, University of Witten Herdecke Helios Hospital Siegburg Siegburg Germany

**Keywords:** cardiac metastasis, malignant embolism, paradox embolism, pulmonal adenocarcinoma

## Abstract

**Background:**

Embolic events play an important role in clinical everyday practice. Malignant arterial embolism is a rare nevertheless often fatal entity for cardiac, cerebral or systemic ischemia, requiring immediate diagnosis and treatment.

**Case:**

This is a case report of a 65 years‐old female, suffering from pulmonal adenocarcinoma, who was hospitalized due to neurological deficits caused by an acute ischemic stroke, followed by anterior myocardial infarction within 3 days. Diagnostic work‐up revealed metastasis of the pulmonal adenocarcinoma in the right atrium and a patent foramen ovale. Histopathological examination of the coronary embolus verified paradoxical arterial embolism of the pulmonal adenocarcinoma into a coronary vessel and consequently cerebral arteries.

**Conclusion:**

The present case underlines the need for (i), consideration of malignant embolism, (ii) histopathological examination of the embolus to determine its etiology, and (iii) interdisciplinary discussion of individual therapeutic and prevention strategies in cancer patients with cerebral, cardiac or systemic embolic events.

## 1 INTRODUCTION

Embolic events play an important role in clinical everyday practice. Besides thromboembolic causes of systemic ischemic diseases, rare entities like malignant arterial embolism may be underestimated. These diagnostic errors often result in improper treatment with oral anticoagulants, ignoring the malignant nature of the embolus, demanding surgical or interventional embolectomy,^1^ which is demonstrated by the following case report.

## 2 CASE

**FIGURE 1:**
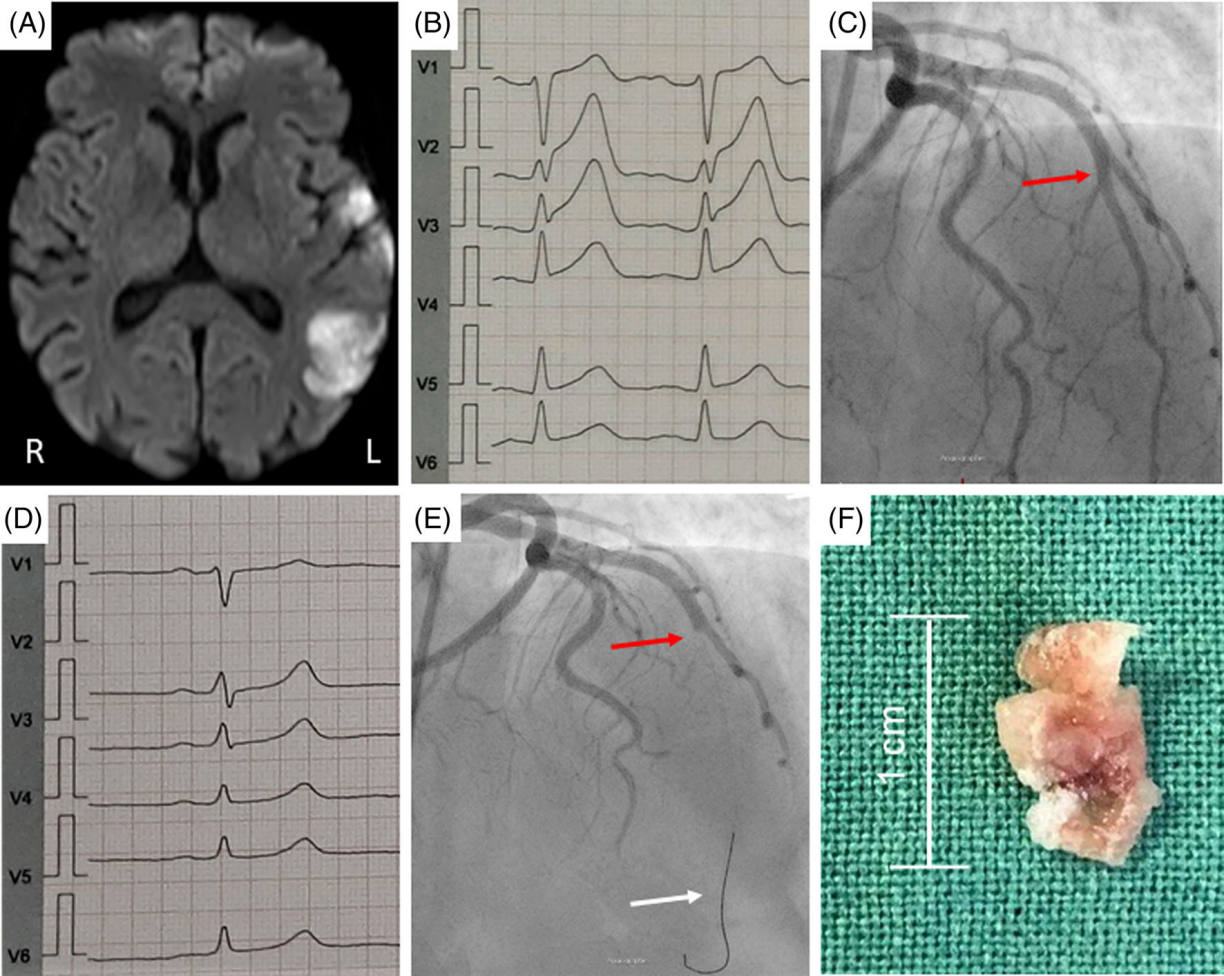
Clinical documentation of patient history. (A) Diffusion‐weighted magnetic resonance imaging (MRI) scan of cerebral ischemia with embolic pattern (L = left, R = right). (B) Electrocardiogram (ECG) on first day of hospital admission. (C) ECG of acute anterior wall infarction with ST‐segment elevation (V1–V4), 3 days after hospitalization. (D) Percutaneous coronary intervention with occlusion of LAD (red arrow) and catheter wire after transcending LAD embolus (white arrow). (E) Successful recanalization of the LAD (red arrow). (F) Malignant embolus of LAD

A 65 years‐old female was admitted to neurological emergency unit with acute onset of aphasia and brachiofacial hemiparesis of the right side (National Institutes of Health Stroke Scale‐score of 3). Cerebral magnetic resonance imaging (MRI) displayed an acute Middle cerebral artery (MCA) infarction (left side), (Figure 1(A)). Pulsed Doppler‐sonography of the carotid‐ and vertebral arteries and transcranial Doppler excluded etiological pathologies. The patient has been suffering from an osseous metastasized pulmonal adenocarcinoma (Pancoast tumor; cT4, cN2, cM0, UICC stage IIIB) since 2015 and was treated first line with Cisplatin/Pemetrexed. Due to progress in disease, treatment was switched to Docetaxel and Nintedanib in 2016. Based on a severe hepatopathy, Nintedanib was exchanged for Nivolumab in 2017. Three months before admission, bone metastasis of the rib was treated by radiotherapy. The MRI at admission revealed incidentally three small cerebral lesions compatible to clinical inapparent metastases. Concomitant diseases were renal failure (stage 3A), degeneration of the lumbar spine, hypercholesterinemia, resection of a cystadenoma of the right ovary in 2016, paroxysmal atrial fibrillation and tumor‐associated thrombosis of the left lower limb in 2016 with subsequent oral anticoagulation with Apixaban. A conservative approach with persistent Apixaban therapy and early rehabilitation was initiated. Three days later, the patient complained about acute chest pain. Electrocardiogram (ECG) showed an extensive anterior myocardial infarction (Figure 1(B),(C)), leading to coronary angiography. An embolic obliteration of the left anterior descending artery (LAD) was successfully treated by primary percutaneous coronary intervention with manual thrombus aspiration (Figure 1(D),(E)), followed by antithrombotic therapy with Acetylsalicylic acid (100 mg/day) and Enoxaparin sodium (0.4 mg/day).

**FIGURE 2:**
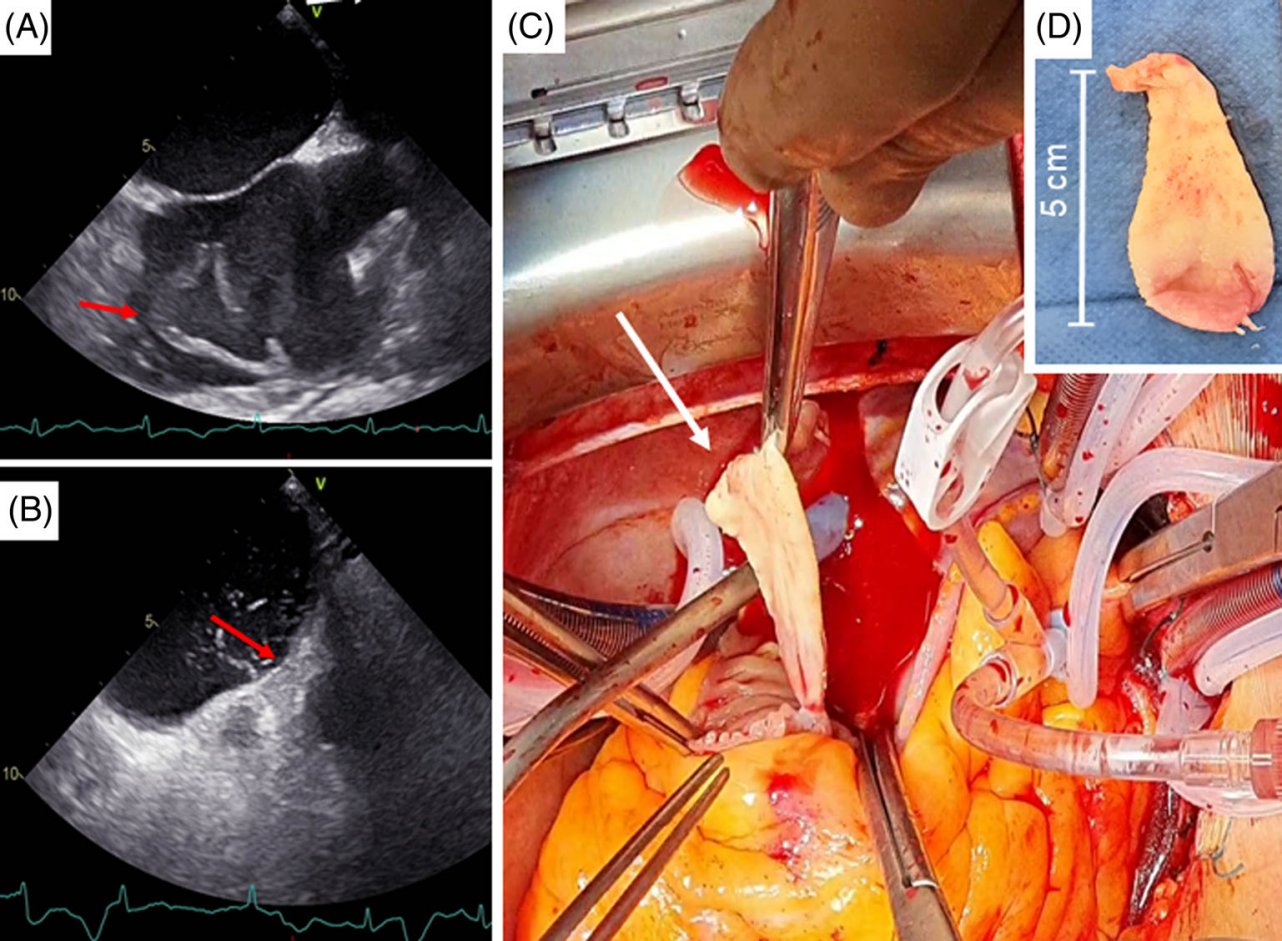
Clinical documentation of findings. (A) Transesophageal echocardiography (TEE) displaying metastasis of the right atrium (red arrow). (B) TEE documented PFO (red arrow). (C) Intraoperative photography of the metastasis in the right atrium (white arrow). (D) Photography of resected metastasis

**FIGURE 3:**
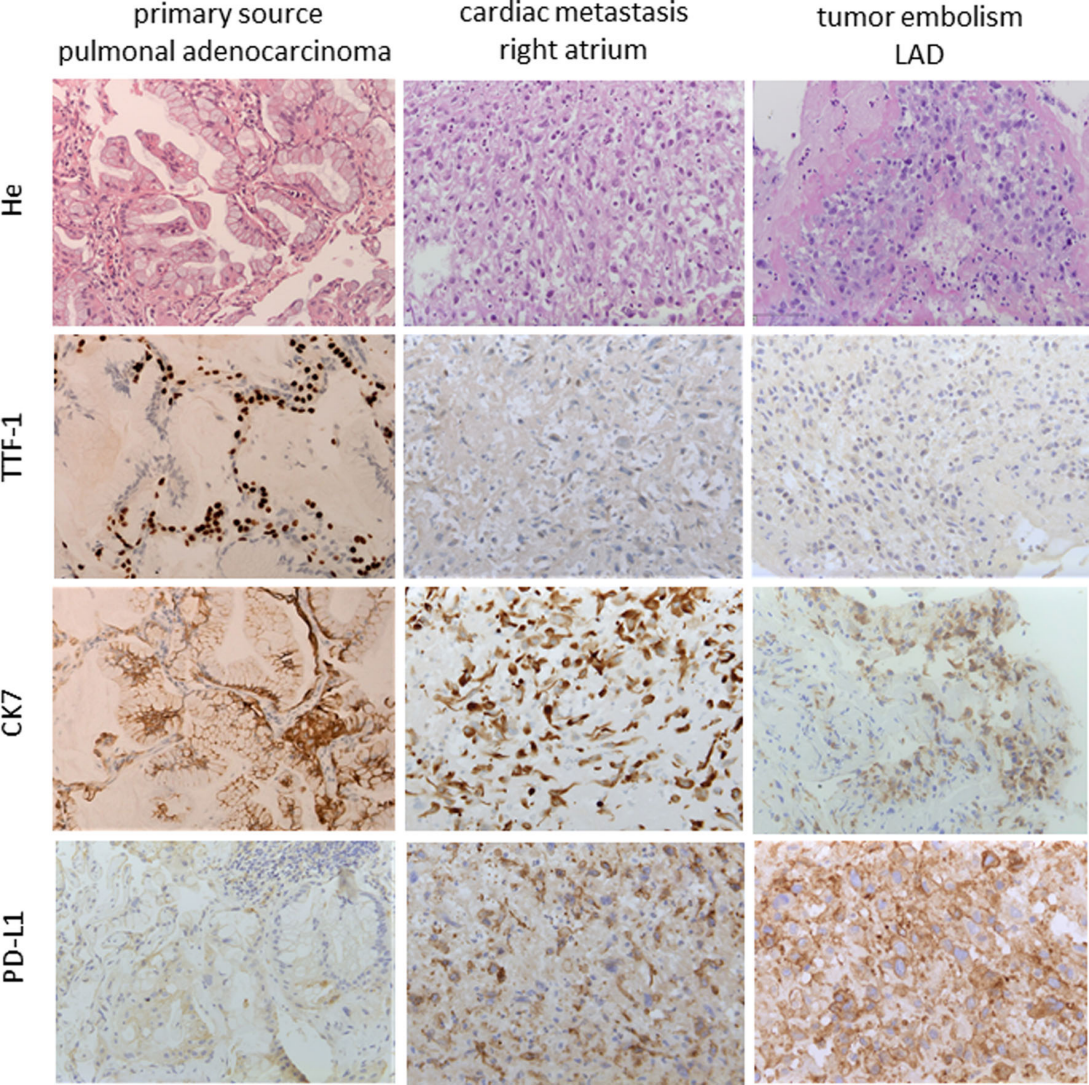
Histomorphological and immunohistochemical examination. Left column: Primary pulmonal adenocarcinoma (2015) with lepidic growth pattern (TTF‐1 negative, CK7 positive, PD‐L1 positive (TP‐Score of 15%)). Middle column: Metastasis of the right atrium (2020). Right column: Malignant embolus of the LAD (2020). The last two displaying coagulated poorly differentiated adenocarcinoma (TTF‐1 negative, CK7 positive, PD‐L1 positive (TP‐Score of 90%)). Magnification: 20×

Histological examination of the LAD‐embolus revealed coagulated poorly differentiated adenocarcinoma. Immunohistochemical analysis yielded a TTF‐1 negative, CK7 positive and PD‐L1 positive (TP‐Score of 90%) tumor cell staining result, corresponding to the primary TTF‐1 negative pulmonary adenocarcinoma with typical lepidic growth pattern (Figure 3). During chemotherapeutical treatment and checkpoint inhibition with Nivolumab, a dedifferentiation and upregulation of PD‐L1 presumably took place. A complementary transesophageal echocardiography discovered a large patent foramen ovale (PFO) and a tumor mass floating in the right atrium with two tumor plugs inserting in the fossa ovalis (Figure 2(A),(B)) as a prove for cerebral and coronary tumor embolization via the PFO.

Fifteen days after hospitalization the patient underwent complete surgical resection of the right atrial tumor mass and PFO closure (Figure 2(C),(D)). Histological examination of the fragile tumor mass verified it as a metastasis of the pulmonal adenocarcinoma (Figure 3). After an initially inconspicuous postoperative course, the patient was noticed with an insufficient increase in consciousness and hemiparesis of the left side during monitoring at the intensive care unit. Computer tomography displayed early infarction sings in the right MCA area through combined occlusion of the right internal carotid artery and MCA and several small cortical lesions with beginning cerebral ischemia and brain edema of the right hemisphere in the manner of pre‐/intraoperative malignant embolism. Due to the lack of promising therapeutic options, the patient received best supportive care until she died 36 h later.

## 3 DISCUSSION

Cerebral and cardiac embolism play an important role in clinical everyday practice and require detailed analysis with respect to their causes. Every tenth patient with ischemic stroke has comorbid cancer due to advances in cancer therapeutics prolonging median survival.^2^ Therefore, besides primary non‐malignant causes like cardiac embolism, vasculitis, coagulation disorders or metabolic‐toxic, rare entities like malignant arterial embolism may be underestimated.

Thromboembolism in general is a common paraneoplastic complication due to a variety of procoagulant properties of the tumor cells; they affect platelet function, which additionally promotes the hypercoagulable state of malignancy.^3^, ^4^, ^5^, ^6^ Further, cancer is associated with alterations of the coagulation and fibrinolytic system^7^, ^8^, ^9^ and endothelial dysfunction and injury promote the progression of atherosclerosis.^10^, ^11^ Further preconditions for malignant arterial embolization are vascular tumor invasion, followed by ejection into systemic circulation.^1^

Common sites of both, malignant and thrombotic emboli, are cerebral arteries, arteries of the lower limb, iliac arteries, and less frequent visceral arteries as well as coronaries or even aortic emboli.^12^ Their etiology must be elucidated as their therapeutic and preventive strategies differ from each other. Thrombotic emboli are treated with and prevented by anticoagulants according to size and location, whereas thromboembolic vessel occlusions are primarily treated with systemic or local thrombolytic therapy.^13^, ^14^ Surgical or interventional embolectomy is the method of choice for malignant arterial embolism but only performed in fulminant thromboembolism.^1^ The ineffectiveness of anticoagulant therapy in the prevention of malignant arterial embolism underlines the importance of histological examination of thromboembolic material in cancer patients. Additionally, some benign (myxoma or papillary fibroelastoma) and in rare cases malignant cardiac tumors (undifferentiated pleomorphic sarcoma, leiomyosarcoma, angiosarcoma or rhabdomyoma) are regularly diagnosed by histological examination of embolic vessel obliterations and are underlying causes of strokes.^15^

A PFO is found in up to 27% of the general population and establishes a direct communication between the right and left atrium generating a route for paradoxical embolism.^16^ Three recent randomized controlled clinical trials (RESPECT, CLOSE and REDUCE) proved that among adults with cryptogenic ischemic stroke, a closure of a PFO, partly in combination with antiplatelet therapy, lowered the rate of recurrent ischemic strokes compared to antiplatelet therapy alone.^17^, ^18^, ^19^ Rigatelli et al. hypothesized that circulating tumor cells reach the brain circulation via two concurrent pathways: First, circulating tumor cells pass the pulmonary filter and reach systemic circulation via pulmonary capillaries; second, they enter systemic circulation via a right to left shunt.^20^ However, in a small prospective pilot study by Levin‐Epstein et al. including nine cancer patients (thyroid‐, breast‐ and upper intestinal carcinoma), was demonstrated a similar prevalence of PFO in patients who developed brain metastases without preceding pulmonal metastasis compared to estimates for the general population.^21^ Further larger studies are needed to reveal if a PFO increases both, the risk of thromboembolic and malignant arterial paradoxical embolism in cancer patients and may therefore be taken into consideration to be closed preventive.

In summary, the present case underlines the need for (i) interdisciplinary discussion, (ii) consideration of malignant embolism, (iii) histopathological examination of the embolus to determine its etiology, and (iv) individual therapeutic and prevention strategy, based on all available information in cancer patients with cerebral or systemic embolic events.

## CONFLICT OF INTEREST

R.B. has received Honoria for Lectures and advisory boards from AbbVie, Amgen, AstraZeneca, Bayer, BMS, Boehringer‐Ingelheim, Illumina, Lilly, Merck‐Serono, MSD, Novartis, Qiagen, Pfizer, Roche, Targos Mol Path. R.B. is a co‐founder and CSO for Targos Mol Path, Kassel (Germany). All other authors have nothing to disclose.

## AUTHOR CONTRIBUTIONS

All authors had full access to the data in the study and take responsibility for the integrity of the data and the accuracy of the data analysis. *Conceptualization*, B.J.W., H.‐P.H., A.J.H., R.B.; *Methodology*, B.J.W., H.‐P.H., A.J.H., M.G.; *Investigation*, B.J.W., H.‐P.H., R.B.; *Formal Analysis*, B.J.W.; *Resources*, B.J.W.; *Writing—Original Draft*, B.J.W., H.‐P.H., R.B.; *Writing—Review & Editing*, B.J.W., H.‐P.H., A.J.H., L.K.H., M.G., N.M., R.B.; *Visualization*, B.J.W., A.J.H., L.K.H., N.M.; *Supervision*, B.J.W., H.‐P.H., L.K.H.; *Data Curation*, B.J.W., H.‐P.H., L.K.H., M.G., N.M., R.B.; *Project Administration*, B.J.W., R.B.; *Validation*, H.‐P.H., L.K.H., M.G., N.M., R.B.

## ETHICAL STATEMENT

Informed consent was obtained from the spouse, as the legal representative of the patient of this case report, to publish the patient data, including potentially identifying individual information in this study.

## Data Availability

The datasets used during the current study are available from the corresponding author on reasonable request.
